# Cardiac Events and Survival in Patients With *EGFR*-Mutant Non–Small Cell Lung Cancer Treated With Osimertinib

**DOI:** 10.1001/jamanetworkopen.2024.48364

**Published:** 2024-12-05

**Authors:** Chien-Yu Lin, Wei-Ting Chang, Po-Lan Su, Chin-Wei Kuo, Jen Yang, Chien-Chung Lin, Sheng-Hsiang Lin

**Affiliations:** 1 Institute of Clinical Medicine, College of Medicine, National Cheng Kung University, Tainan, Taiwan; 2 Department of Internal Medicine, National Cheng Kung University Hospital, College of Medicine, National Cheng Kung University, Tainan, Taiwan; 3 Division of Cardiology, Department of Internal Medicine, Chi-Mei Medical Center, Tainan, Taiwan; 4 Department of Biotechnology, Southern Taiwan University of Science and Technology, Tainan; 5 Department of Biomedical Engineering, College of Engineering, National Cheng Kung University, Tainan, Taiwan; 6 Department of Medical Imaging, National Cheng Kung University Hospital, College of Medicine, National Cheng Kung University, Tainan, Taiwan; 7 Institute of Molecular Medicine, College of Medicine, National Cheng Kung University, Tainan, Taiwan; 8 Tainan Hospital, Ministry of Health and Welfare, Tainan, Taiwan; 9 Biostatistics Consulting Center, National Cheng Kung University Hospital, College of Medicine, National Cheng Kung University, Tainan, Taiwan; 10 Department of Public Health, College of Medicine, National Cheng Kung University, Tainan, Taiwan

## Abstract

**Importance:**

Although it has been reported that osimertinib mesylate provides better survival benefits compared with first- or second-generation epidermal growth factor receptor tyrosine kinase inhibitors (EGFR TKIs), it remains unclear whether osimertinib is associated with more cancer therapy–related cardiac events (CTRCEs) compared with other EGFR TKIs, as does the extent of the association these adverse effects may have with overall survival. This issue is particularly critical due to the high prevalence of *EGFR* variants within Asian populations, including that of Taiwan.

**Objective:**

To compare CTRCEs and their association with survival in patients treated with osimertinib vs other EGFR TKIs.

**Design, Setting, and Participants:**

This cohort study was conducted at the National Cheng Kung University Hospital, a college hospital and tertiary academic referral center in Taiwan. The median follow-up duration was 23.2 (IQR, 15.2-31.5) months. A total of 401 patients with *EGFR*-mutant non–small cell lung cancer (NSCLC) beginning treatment with EGFR TKIs from September 1, 2019, to July 31, 2022, were retrospectively analyzed. CTRCEs included newly emerging arrhythmias, valvular heart diseases (moderate and more), myocardial infarction, and heart failure and were analyzed after adjusting for age, sex, smoking, alcohol consumption, body mass index, cardiovascular comorbidities, thoracic radiotherapy, and cardiovascular medications. Follow-up was completed January 31, 2024.

**Exposure:**

Osimertinib.

**Main Outcomes and Measures:**

The Cox proportional hazards model was used to estimate CTRCEs in patients treated with osimertinib or other EGFR TKIs. Considering that death can lower the incidence of CTRCEs, the competing risk method was used to calculate CTRCEs after adjusting for potential confounders. Multivariable Cox proportional hazard regression analysis for overall survival was used to explore whether CTRCEs were independently associated with overall survival.

**Results:**

Among the 401 patients (253 [63.1%] female; mean [SD] age, 69.2 [11.3] years), 195 (48.6%) treated with osimertinib were matched with 206 (51.4%) treated with other EGFR TKIs. Occurrence of CTRCEs in patients receiving osimertinib was significantly higher compared with patients treated with other EGFR TKIs (29 [14.9%] vs 9 [4.4%]; hazard ratio [HR], 3.37; 95% CI, 1.56-7.26; *P* = .002). After adjustment for relevant cardiovascular risk factors, the HR of CTRCEs was significantly higher in the group treated with osimertinib (adjusted subdistribution HR, 4.00; 95% CI, 1.81-8.85; *P* < .001). In addition, CTRCEs were independently associated with overall survival (HR, 4.02; 95% CI, 2.44-6.63; *P* < .001).

**Conclusions and Relevance:**

In this cohort study of patients with *EGFR*-mutant NSCLC, osimertinib was associated with a higher incidence of CTRCEs compared with other EGFR TKIs; CTRCEs were independently associated with overall survival. These findings highlight the need for ongoing cardiac monitoring in these patients, regardless of preexisting cardiac risk factors.

## Key Points

**Question:**

Do patients diagnosed with *EGFR*-mutant non–small cell lung cancer (NSCLC) receiving osimertinib have higher cancer therapy–related cardiac events (CTRCEs) compared with those receiving other epidermal growth factor receptor tyrosine kinase inhibitors (EGFR TKIs)?

**Findings:**

In a cohort study of 401 patients in Taiwan with *EGFR*-mutant NSCLC treated with EGFR TKIs, those receiving osimertinib experienced significantly more CTRCEs compared with other EGFR TKIs. In addition, CTRCEs were found to be independently associated with overall survival.

**Meaning:**

These findings suggest the need for close cardiac monitoring of patients receiving osimertinib.

## Teaser

This cohort study examines whether osimertinib is associated with a higher incidence of cardiac toxic effects compared with earlier-generation epidermal growth factor receptor tyrosine kinase inhibitors among patients in Taiwan with *EGFR*-mutant non–small cell lung cancer.

## Introduction

Lung cancer stands as a leading cause of cancer-related deaths worldwide, posing significant challenges in patient outcomes. While there have been advances in treatment options, the 5-year survival rate for patients with advanced non–small cell lung cancer (NSCLC) remains disappointingly low, often below 10%.^1,2^ Epidermal growth factor receptors (EGFRs) take center stage as the key drivers of NSCLC.^3^ Treatment predominantly involves tyrosine kinase inhibitors (TKIs) in patients with *EGFR* variants.^4,5,6,7,8,9,10^ Based on guidelines from the National Comprehensive Cancer Network, a third-generation EGFR TKI, osimertinib, has emerged as the favored choice among EGFR TKIs for initial treatment.^11^ This recommendation stems from the overall survival (OS) benefit demonstrated in the FLAURA study when compared with either erlotinib hydrochloride or gefitinib.^10^ For patients developing the T790M mutation after initial EGFR TKI treatments, wherein around 60% experience progression, osimertinib has emerged as the recommended second-line treatment based on evidence from the AURA3 trial.^12^ Recent studies have further demonstrated that osimertinib exhibits a notable improvement in disease-free survival compared with placebo in patients with *EGFR*-mutant stages IB to IIIA NSCLC following complete tumor resection, with or without adjuvant chemotherapy.^13^

The FLAURA study demonstrated that adverse events of grade 3 or higher occurred less frequently in the osimertinib group compared with the standard EGFR-TKI group.^14^ However, concerns exist regarding the underreporting of patient symptoms in clinical trials.^15,16^ Previous research by Chang et al^17^ identified an association between EGFR-TKI use and an increased likelihood of cardiac and cerebrovascular events. Osimertinib-associated cardiac toxic effects, including arrhythmia, cardiac dysfunction, progression of valvular heart disease, and myocardial infarction, have been reported.^18,19^ However, whether osimertinib carries a higher incidence of cardiac toxic effects compared with other EGFR TKIs remains a topic of debate. Many studies have overlooked the underlying cardiovascular health of patients and the impact of death as a competing risk in their evaluations.^20,21,22^ To address these uncertainties, we conducted a retrospective cohort study that considers patients’ individual cardiovascular risk and treats death as a competing risk. Our study’s aim was to examine whether osimertinib was associated with a higher incidence of cardiac toxic effects compared with earlier-generation EGFR TKIs. Additionally, we explored the potential association of osimertinib-associated cardiac toxic effects with patient mortality rates.

## Methods

### Patients and Study Design

This was a retrospective cohort study conducted at the National Cheng Kung University Hospital (NCKUH), a college hospital and tertiary academic referral center serving the 1.86 million inhabitants of Tainan, Taiwan. This study was approved by the Review Board and Ethics Committee of NCKUH, which waived the requirement for informed consent because of the retrospective nature of the study. All data were anonymized in compliance with approved guidelines and the Declaration of Helsinki.^23^ This study adheres to the reporting guidelines outlined in the Strengthening the Reporting of Observational Studies in Epidemiology (STROBE) protocol.

 Electronic medical records were reviewed for all patients 20 years or older with a diagnosis of histologically and cytologically confirmed NSCLC harboring an *EGFR* variation who began treatment with EGFR TKIs between September 1, 2019, and July 31, 2022, at NCKUH. Patients were excluded if their medical records were unobtainable or incomplete or if they had used an EGFR TKI for less than 2 weeks. A control group consisting of patients with *EGFR*-mutant NSCLC who had received first- or second-generation agents (erlotinib, gefitinib, afatinib, and dacomitinib) was matched by age, sex, smoking, alcohol consumption, body mass index, cardiovascular comorbidities (type 2 diabetes, hypertension, hyperlipidemia, coronary artery disease, heart failure, chronic kidney disease, and arrhythmia), thoracic radiotherapy, and cardiovascular medications (angiotensin-converting enzyme inhibitors and/or angiotensin receptor blockers, β-blockers, antiplatelet agents, anticoagulants, statins, and antiarrhythmic drugs) using stabilized inverse probability of treatment weighting (IPTW) with the experimental group, namely those patients who received osimertinib.

### Study End Point

The primary end point of the study was the occurrence of cancer therapy–related cardiac events (CTRCEs), defined as newly emerging arrhythmias (including symptomatic supraventricular tachycardia, atrial fibrillation, symptomatic ventricular arrhythmias [including symptomatic ventricular premature complexes], nonsustained ventricular tachycardia, ventricular tachycardia, and ventricular fibrillation, and atrioventricular block), valvular heart diseases (moderate and more severe), myocardial infarction, and heart failure defined by the 2022 European Society of Cardiology guidelines on cardio-oncology as a greater than 10% decline in left ventricular ejection fraction (LVEF) from the baseline, or an LVEF below 50%, occurring 2 months after the use of EGFR TKIs. The secondary end point was OS, defined as the period from the initiation of EGFR TKI as first-line treatment to the date of death due to any cause. The median follow-up time was 23.2 (IQR, 15.2-31.5) months, and the last follow-up appointment was January 31, 2024.

### Statistical Analyses

Continuous variables are expressed as means and SDs, while categorical variables are presented as counts and percentages. To mitigate selection bias due to variations in clinical characteristics, we used propensity score analysis with the stabilized IPTW method.^24^ Propensity scores estimate the likelihood of receiving a treatment based on baseline characteristics. In this study, propensity scores for the use of osimertinib were calculated using multivariate logistic regression. The factors considered included age, sex, smoking status, alcohol use, body mass index, thoracic radiotherapy, pretreatment cardiovascular comorbidities, and cardiovascular medications used. The mean stabilized weight of our model (0.997) is very close to 1, ensuring the adequacy and balance of the propensity score model.^25^ Absolute standardized mean difference (ASMD) was used to assess the comparability of clinical features between the 2 groups. An ASMD value of 0.1 indicates that there is no significant difference between the 2 groups in terms of the distribution of the variables being examined. This metric was computed by dividing the mean or proportion of a variable by the pooled estimate of the SD of that variable.

Afterward, we used the multivariate Cox proportional hazards model to examine the association between the CTRCEs and various treatments by calculating the hazard ratios (HRs) and their 95% CIs. We used the scaled Schoenfeld residuals method and found that the *P* values were not significant, which indicates that our Cox regression model meets the proportional hazards assumption. Moreover, recognizing that death might impact the occurrence of cardiovascular events, we used the competing risk method (subdistribution HR) to compute the HRs of CTRCE derived from the Cox regression model. All confounders were well matched after IPTW (with ASMDs all less than 0.1), and adjustment for confounders was also conducted. Additionally, to explore whether CTRCE was independently associated with overall survival, we performed multivariable Cox proportional hazard regression analysis for overall survival, incorporating programming statements to accommodate CTRCE as a time-dependent covariate. The cumulative incidence function was used to visualize the results for both the primary outcome event and competing risk events such as death. Differences between these events were assessed using the Gray test. Statistical significance was determined using 2-sided *P* < .05. Data analyses were conducted using SAS, version 9.4 for Windows (SAS Institute Inc), and Python, version 3.10 (Python Software Foundation).

## Results

### Patient Characteristics

From September 1, 2019, to July 31, 2022, we identified 401 patients with NSCLC beginning treatment with EGFR TKIs. Of those, 195 patients (48.6%) treated with osimertinib were matched with 206 patients (51.4%) who received other EGFR TKIs (eFigure 1 in Supplement 1). The mean (SD) age was 69.2 (11.3) years, with 253 female (63.1%) and 148 male patients (36.9%), and most were nonsmokers (331 [82.5%]). A total of 169 patients (weighted 42.4%) had a history of hypertension, while 80 (weighted 20.0%) had a diagnosis of diabetes or hyperlipidemia. Approximately one-fifth of these patients were prescribed angiotensin-converting enzyme inhibitors and/or angiotensin receptor blockers (81 [weighted 20.2%]) or statins (76 [weighted 19.0%]), and one-tenth received β-blockers (55 [weighted 13.7%]). Moreover, 112 patients (weighted 27.9%) underwent thoracic radiotherapy (Table 1).

**Table 1.** Baseline Characteristics of Patients With *EGFR*-Mutant NSCLC Treated With Osimertinib or Other EGFR TKIs Before and After Stabilized IPTW^a^

Characteristic	Before IPTW, No. (%)				After stabilized IPTW, No. (%)					
	Osimertinib use (n = 195)	Other EGFR TKI use (n = 206)	*P* value	ASMD		Osimertinib use	Other EGFR TKI use		*P* value	ASMD
Age, mean (SD), y	67.4 (11.6)	70.9 (10.7)	.001	0.32		69.0 (11.5)		69.2 (10.7)	.87	0.02
Sex										
Male	70 (35.9)	78 (37.9)	.68	0.04		70 (33.9)		74 (35.8)	.92	0.01
Female	125 (64.1)	128 (62.1)				125 (66.1)		132 (64.2)		
Smoking	30 (14.6)	40 (19.4)	.28	0.11		35 (17.0)		37 (17.9)	.95	0.01
Alcohol use	10 (4.9)	10 (4.9)	.89	0.01		10 (5.1)		11 (5.2)	.92	0.01
BMI, mean (SD)	24.0 (3.5)	22.3 (3.8)	.005	0.29		23.6 (3.4)		23.6 (4.0)	.90	0.02
Thoracic RT	58 (28.2)	54 (26.2)	.43	0.08		54 (26.3)		58 (28.3)	.96	0.01
Comorbidities										
Diabetes	35 (17.0)	45 (21.8)	.32	0.10		39 (18.9)		43 (20.7)	.90	0.01
Hypertension	79 (38.4)	90 (43.7)	.51	0.06		79 (38.5)		86 (42.0)	.87	0.02
Hyperlipidemia	39 (18.9)	41 (19.9)	.98	0.002		36 (17.5)		40 (19.3)	.86	0.02
Coronary artery disease	10 (4.9)	15 (7.3)	.37	0.09		11 (5.4)		12 (6.0)	.91	0.01
Heart failure	1 (0.5)	5 (2.4)	.11	0.16		2 (0.9)		3 (1.5)	.67	0.05
Chronic kidney disease	6 (2.9)	9 (4.4)	.49	0.07		6 (3.1)		8 (3.8)	.82	0.02
Arrhythmia	7 (3.4)	13 (6.3)	.21	0.13		9 (4.3)		11 (5.2)	.78	0.03
Cardiovascular medications										
ACEIs and/or ARBs	38 (18.5)	43 (20.9)	.72	0.03		37 (18.0)		41 (19.7)	.91	0.01
β-Blockers	25 (12.1)	30 (14.6)	.61	0.05		24 (11.7)		27 (13.3)	.81	0.02
Antiplatelet agents	12 (5.8)	27 (13.1)	.02	0.24		16 (7.9)		19 (9.4)	.75	0.03
Anticoagulants	4 (1.9)	6 (2.9)	.58	0.06		4 (2.1)		5 (2.4)	.91	0.01
Statins	37 (18.0)	39 (18.9)	.99	0.001		34 (16.7)		38 (18.4)	.88	0.02
Antiarrhythmic drugs	7 (3.4)	8 (3.9)	.87	0.02		7 (3.5)		8 (3.8)	.98	0.003

Abbreviations: ACEIs, angiotensin-converting enzyme inhibitors; ARBs, angiotensin receptor blockers; ASMD, absolute standardized mean difference; BMI, body mass index (calculated as the weight in kilograms divided by the height in meters squared); EGFR TKI, epidermal growth factor receptor tyrosine kinase inhibitor; IPTW, inverse probability of treatment weighting; NSCLC, non–small cell lung carcinoma; RT, radiotherapy.

^a^ Mean stabilizing weight was 0.997.

### Comparison of CTRCEs Between Osimertinib and Other EGFR TKI Treatment

Occurrence of CTRCEs in patients receiving osimertinib was significantly higher compared with patients treated with other EGFR TKIs (29 [14.9%] vs 9 [4.4%]; HR, 3.37; 95% CI, 1.56-7.26; *P* = .002) (Table 2). Given that some patients may die before experiencing CTRCE, we accounted for mortality as a competing risk. Additionally, we adjusted for relevant cardiovascular risk factors. Similarly, we found significantly increased CTRCEs in those receiving osimertinib compared with those receiving other EGFR TKIs (adjusted subdistribution HR [sHR], 4.00; 95% CI, 1.81-8.85; *P* < .001) (Table 2). Furthermore, the cumulative incidence of CTRCE in the osimertinib group was significantly higher than in the group receiving other EGFR TKIs (Gray test *P* < .001) (Figure). The osimertinib group had a significantly higher likelihood of newly developed arrhythmia compared with the group receiving other EGFR TKIs, in addition to an increased likelihood of heart failure (eTable in Supplement 1).

**Table 2.** CTRCEs With Treatment of *EGFR*-Mutant NSCLC After IPTW

Treatment	CTRCEs, No. (%) of patients	HR (95% CI)	*P* value	sHR (95% CI)	*P* value	Adjusted sHR (95% CI)^a^	*P* value
Osimertinib use (n = 195)	29 (14.9)	3.37 (1.56-7.26)	.002	3.74 (1.76-7.97)	.001	4.00 (1.81-8.85)	<.001
Other EGFR TKI use (n = 206)	9 (4.4)	1 [Reference]		1 [Reference]		1 [Reference]	

Abbreviations: CTRCEs, cancer therapy–related cardiac events; EGFR TKI, epidermal growth factor receptor tyrosine kinase inhibitor; HR, hazard ratio; IPTW, inverse probability of treatment weighting; NSCLC, non–small cell lung carcinoma; sHR, subdistribution HR.

^a^ Model was adjusted for age, sex, thoracic radiotherapy, cardiovascular medication (angiotensin-converting enzyme inhibitors and/or angiotensin receptor blockers, β-blockers, antiplatelet agents, anticoagulants, statins, or antiarrhythmia drugs), and comorbidities (coronary artery disease, hypertension, diabetes, hyperlipidemia, heart failure, chronic kidney disease, or arrhythmia).

**Figure.**
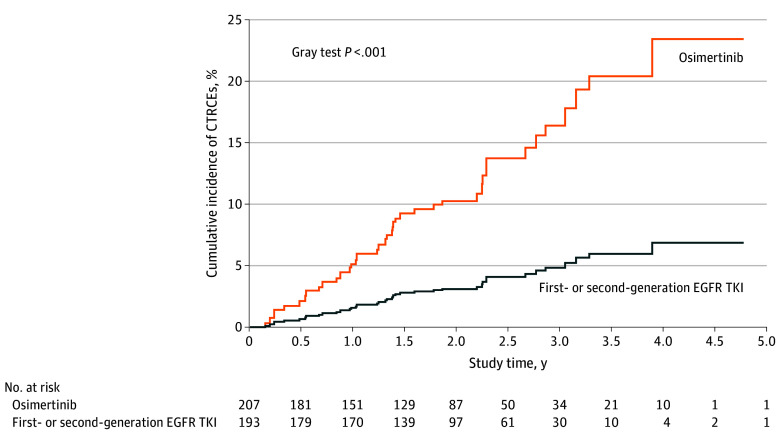
Accumulating Incidences of Cancer Therapy–Related Cardiac Events (CTRCEs) Among Patients With Non–Small Cell Lung Cancer Receiving Osimertinib vs Other Epidermal Growth Factor Receptor Tyrosine Kinase Inhibitors (EGFR TKIs) The individuals at risk in each group at intervals along the x-axis represent the estimated number of people due to weighting.

### Subgroup Analysis of CTRCEs Among Patients With vs Without Osimertinib Use

Some studies^18,26^ have suggested that CTRCEs attributed to osimertinib predominantly occur in patients with preexisting cardiovascular disease (CVD) or cardiovascular risk factors. Therefore, we conducted a subgroup analysis in patients without CVD or cardiovascular risk factors and discovered that the hazard of CTRCEs was still significantly increased in osimertinib users compared with other EGFR TKI users (eFigure 3 in Supplement 1).

Furthermore, we divided patients into 2 groups based on low (0-2) and high (≥3) numbers of preexisting CVD or cardiovascular risk factors. In the low-risk group, patients treated with osimertinib showed a significantly higher hazard of CTRCEs after adjustment (adjusted sHR, 8.78; 95% CI, 1.65-46.70; *P* = .01) (Table 3). Similarly, the cumulative incidence of CTRCEs in this subgroup was significantly higher in the osimertinib group compared with the group receiving other EGFR TKIs (Gray test *P* < .001) (eFigure 2 in Supplement 1).

**Table 3.** Subgroup Analysis of CTRCEs Between Patients With *EGFR*-Mutated NSCLC and Different Preexisting CVD or Cardiovascular Risk Factors

No. of preexisting CVD or cardiovascular risk factors^a^	Treatment, patients with CTRCES/Total No. of patients (%)		HR (95% CI)	*P* value	sHR (95% CI)	*P* value	Adjusted sHR (95% CI)^b^	*P* value
	Osimertinib	Other EGFR TKI						
0-2	17/119 (14.29)	2/116 (1.72)	7.40 (1.52-36.15)	.01	8.83 (1.82-42.97)	.007	8.78 (1.65-46.70)	.01
≥3	12/76 (15.79)	7/88 (7.95)	2.06 (0.80-5.27)	.13	2.17 (0.87-5.43)	.10	2.33 (0.65-8.40)	.20

Abbreviations: CTRCEs, cancer therapy–related cardiac events; CVD, cardiovascular disease; EGFR TKI, epidermal growth factor receptor tyrosine kinase inhibitor; HR, hazard ratio; NSCLC, non–small cell lung carcinoma; sHR, subdistribution HR.

^a^ Includes smoking, alcohol use, body mass index (calculated as the weight in kilograms divided by the height in meters squared) greater than 24, thoracic radiotherapy, comorbidities (diabetes, hypertension, hyperlipidemia, coronary artery disease, heart failure, chronic kidney disease, and arrhythmia), and cardiovascular medication use (angiotensin-converting enzyme inhibitors [ACEIs] and/or angiotensin receptor blockers [ARBs], β-blockers, antiplatelet agents, anticoagulants, statins, and antiarrhythmic drugs).

^b^ Adjusted for age, sex, thoracic radiotherapy, cardiovascular medication (ACEIs and/or ARBs, β-blockers, antiplatelet agents, anticoagulants, statins, and antiarrhythmia drugs), and comorbidities (coronary artery disease, hypertension, diabetes, hyperlipidemia, heart failure, chronic kidney disease, and arrhythmia).

### Identification of VariablesD Independently Associated With Overall Survival

To assess whether CTRCEs and/or other variables were independently associated with OS in patients treated with EGFR TKIs, we applied univariate and multivariable Cox proportional hazards regression analysis. This involved using a programming statement to fit a model that includes CTRCEs as a time-dependent covariate. This analysis revealed that, in the multivariable analysis, CTRCEs were independently associated with OS (HR, 4.02; 95% CI, 2.44–6.63; *P* < .001) (Table 4). Other variables independently associated with OS include age, stage, brain metastasis, and performance status score.

**Table 4.** Assessment of Variables Independently Associated With Overall Survival Through Cox Proportional Hazard Regression Analysis

Variable	Univariate Analysis		Multivariable Analysis	
	HR (95% CI)	*P* value	HR (95% CI)	*P* value
CTRCEs^a^	4.18 (2.54-6.85)	<.001	4.02 (2.44-6.63)	<.001
Age (≥65 vs <65 y)	1.56 (1.14-2.13)	.005	1.45 (1.05-2.02)	.03
Sex (male vs female)	1.31 (0.98-1.74)	.07	NA	NA
Variant (uncommon vs common)	1.64 (0.91-2.96)	.10	NA	NA
Morphology (adenocarcinoma vs nonadenocarcinoma)	2.12 (0.87-5.15)	.10	NA	NA
Stage (IV vs I-III)	2.74 (1.56-4.82)	<.001	2.55 (1.27-5.11)	.008
Brain metastasis (with vs without)	1.95 (1.47-2.59)	<.001	1.91 (1.39-2.60)	<.001
Propensity score (≥3 vs <3)	4.59 (2.15-9.81)	<.001	4.58 (2.12-9.88)	<.001
Smoking (smoker vs nonsmoker)	1.21 (0.84-1.75)	.30	NA	NA

Abbreviations: CTRCEs, cancer therapy–related cardiac events; HR, hazard ratio; NA, not applicable.

^a^ Time-dependent covariate, using programming statement to fit model with time-dependent covariate.

## Discussion

Despite the extended survival observed in patients with EGFR variations treated with osimertinib compared with gefitinib or erlotinib,^10^ long-term outcomes may be compromised by significant cardiac risks,^19,22^ particularly given the high prevalence of *EGFR* variants (about 50%) in Asian populations.^27^ A retrospective study involving a Japanese population reported grade 3 or higher cardiac adverse events in nearly 5% of patients,^18^ a rate higher than that observed in clinical trials.^12,13,14^ The present study revealed an even higher incidence of cardiovascular toxic effects, at 14.9%, including newly emerging arrhythmias, valvular heart diseases, myocardial infarction, and heart failure, aligning with a case series that showed a more severe clinical phenotype in the osimertinib group than previously recognized.^28^ Furthermore, considering mortality as a competing risk, our study found that osimertinib was associated with a higher hazard of CTRCEs than other EGFR TKIs, with a significantly greater cumulative incidence of CTRCEs in the osimertinib group. Importantly, CTRCE emerged as being independently associated with OS in *EGFR*-mutant NSCLC, and this hazard remained unassociated with preexisting CVD or traditional risk factors, underscoring the necessity for vigilant cardiac monitoring in this patient population.

Osimertinib, a first-line treatment for *EGFR*-mutated NSCLC, often leads to adverse effects like rashes, diarrhea, and dry skin, with serious adverse effects in 22% of patients vs 25% of patients receiving other EGFR-TKIs, and interstitial lung disease in 2.2% vs 1.4%.^14^ Analyses do not link osimertinib to heart failure, yet reduced LVEF has been noted in patients with cardiac risk.^20^ Other studies^18,20^ suggest higher rates of cardiac toxic effects, potentially not dose dependent, than clinical trials report. A Stanford study of 862 patients^28^ found 17 cases of cardiomyopathy with a 70.6% mortality rate, leading to significant treatment changes, and 9 hospitalizations for LVEF reduction, showing severe cardiac issues. However, some studies^20,29,30^ did not consider preexisting cardiovascular conditions, which yielded inconsistent results. Moreover, preexisting CVD has been associated with cardiac events in patients receiving osimertinib, with reports of acute heart failure even in those without risk factors.^31^ We collected cardiovascular risk data such as smoking, body mass index, CVD, medication history, and thoracic radiotherapy exposure, using IPTW to ensure comparability and to account for death as a competing risk. Our analysis found that the osimertinib group had a significantly higher hazard of CTRCEs (subdistribution HR, 3.74; *P* = .001). Notably, even lower-risk patients (having ≤2 risk factors) treated with osimertinib had a significantly higher CTRCE incidence. This highlights the need for careful monitoring of cardiac adverse effects, even in patients without preexisting cardiovascular conditions or risk factors.

In a recent cohort study including 862 patients prescribed with osimertinib for *EGFR*-mutant NSCLC of any stage,^28^ for patients with cardiotoxic effects, the time from LVEF decrease to death was very short, with a median of only 3.2 (IQR, 1.7-18.4) months. Therefore, we aimed to determine whether CTRCEs are associated with patient mortality and diminished survival benefit of EGFR TKIs. Cox proportional hazards regression analysis was used to assess whether CTRCEs were independently associated with OS in patients treated with EGFR TKIs (HR, 4.02; *P* < .001). Although CTRCEs may impact OS, the cardiac toxic effects induced by osimertinib are generally classified as a type II cancer therapy–related cardiac dysfunction, which is considered dose independent and reversible.^19,32,33^ This implies that the impact of cardiac toxic effects induced by osimertinib on OS can be shortened through early detection and timely dose reduction or discontinuation.

Despite the underlying mechanisms of osimertinib-induced cardiac toxic effects remaining only partially understood, several potential mechanisms have been suggested. A preclinical study^34^ demonstrated that both osimertinib and its metabolite, AZ5104, exhibited off-target activity against erb-b2 receptor tyrosine kinase 2 (ERBB2; previously human epidermal growth factor receptor-2) and displayed greater efficacy against ERBB2 compared with afatinib, a pan-ERBB2 family TKI. ERBB2-targeted therapies such as trastuzumab are recognized medications linked to elevated occurrences of both symptomatic and asymptomatic cardiac dysfunction.^35^ Further research is required to ascertain the association between osimertinib-induced cardiac toxic effects and its inhibition of the ERBB2 pathway. Moreover, osimertinib has been reported to simultaneously block the hERG potassium channels, a phenomenon associated with QT prolongation; cardiac sodium channel Nav1.5, which is associated with slowed conduction; and L-type calcium ion channels, which trigger excitation-contraction coupling, modulate the action potential shape, and are involved in cardiac arrhythmia. This can lead to adverse cardiovascular events such as heart failure and QT prolongation in some patients with NSCLC.^36,37,38^

### Limitations

Our study has some limitations. First, this is a retrospective study. Second, the 2022 European Society of Cardiology Guidelines on cardio-oncology recommended that echocardiography every 3 months should be considered for patients undergoing osimertinib therapy; however, our patients began their treatment between September 1, 2019, and July 31, 2022, and consequently not all patients had routine echocardiography. Therefore, patients with consistent monitoring may introduce detection bias. Third, there is a lack of information regarding the impact of osimertinib-induced cardiac toxic effects on quality of life. Fourth, the limited number of cardiac events (29 for osimertinib and 9 for other EGFR TKIs) might restrict definitive conclusions. However, we found a higher cumulative incidence of CTRCEs in the osimertinib group compared with the other EGFR TKI group. Even patients with low cardiovascular risk while receiving osimertinib had a higher hazard of CTRCEs. These findings provide important new insights.

## Conclusions

For patients diagnosed with *EGFR*-mutant NSCLC, this cohort study found that individuals treated with osimertinib had a higher incidence of CTRCEs compared with those who received other EGFR TKIs. Moreover, CTRCEs were independently associated with OS. These findings highlight the importance of cardiac monitoring to evaluate cardiovascular toxic effects in these patients, irrespective of preexisting cardiac risk factors.

## Notes

Supplement 1. **eFigure 1.** Flowchart of the Study Design **eFigure 2.** Accumulating Incidences of Cancer Therapy–Related Cardiac Events (CTRCEs) Among Patients With Non–Small Cell Lung Cancer **eFigure 3.** Subgroup Analysis of Cancer Therapy–Related Cardiac Events (CTRCEs) Between Patients With Non–Small Cell Lung Cancers (NSCLC) Receiving Osimertinib vs Other Epidermal Growth Factor Receptor (EGFR) Tyrosine Kinase Inhibitors (TKIs) **eTable.** Number and sHR of Individual CTRCEs of Patients With *EGFR*-Variant NSCLC Treated With or Without Osimertinib After IPTW

Supplement 2.
Data Sharing Statement
