# Supplementary material for: Cardiac Events and Survival in Patients With EGFR-Mutant Non–Small Cell Lung Cancer Treated With Osimertinib
Source: JAMA Netw Open. 2024 Dec 5;7(12):e2448364. doi: 10.1001/jamanetworkopen.2024.48364 (PMC11621985; doi:10.1001/jamanetworkopen.2024.48364)
Supplement: Supplement 1. — eFigure 1. Flowchart of the Study Design eFigure 2. Accumulating Incidences of Cancer Therapy–Related Cardiac Events (CTRCEs) Among Patients With Non–Small Cell Lung Cancer eFigure 3. Subgroup Analysis of Cancer Therapy–Related Cardiac Events (CTRCEs) Between Patients With Non–Small Cell Lung Cancers (NSCLC) Receiving Osimertinib vs Other Epidermal Growth Factor Receptor (EGFR) Tyrosine Kinase Inhibitors (TKIs) eTable. Number and sHR of Individual CTRCEs of Patients With EGFR-Variant NSCLC Treated With or Without Osimertinib After IPTW [file jamanetwopen-e2448364-s001.pdf]

Supplementary Online Content

Lin CY, Chang WT, Su PL, et al. Cardiac events and survival in patients with *EGFR*-mutant non–small cell lung cancer treated with osimertinib. *JAMA Netw Open*. 2024;7(12):e2448364. doi:10.1001/jamanetworkopen.2024.48364

eFigure 1. Flowchart of the Study Design
eFigure 2. Accumulating Incidences of Cancer Therapy–Related Cardiac Events (CTRCEs) Among Patients With Non–Small Cell Lung Cancer
eFigure 3. Subgroup Analysis of Cancer Therapy–Related Cardiac Events (CTRCEs) Between Patients With Non–Small Cell Lung Cancers (NSCLC) Receiving Osimertinib vs Other Epidermal Growth Factor Receptor (EGFR) Tyrosine Kinase Inhibitors (TKIs)
eTable. Number and sHR of Individual CTRCEs of Patients With EGFR-Variant NSCLC Treated With or Without Osimertinib After IPTW

This supplementary material has been provided by the authors to give readers additional information about their work.

© 2024 Lin C-Y et al. *JAMA Network Open*.

eFigure 1. Flowchart of the Study Design

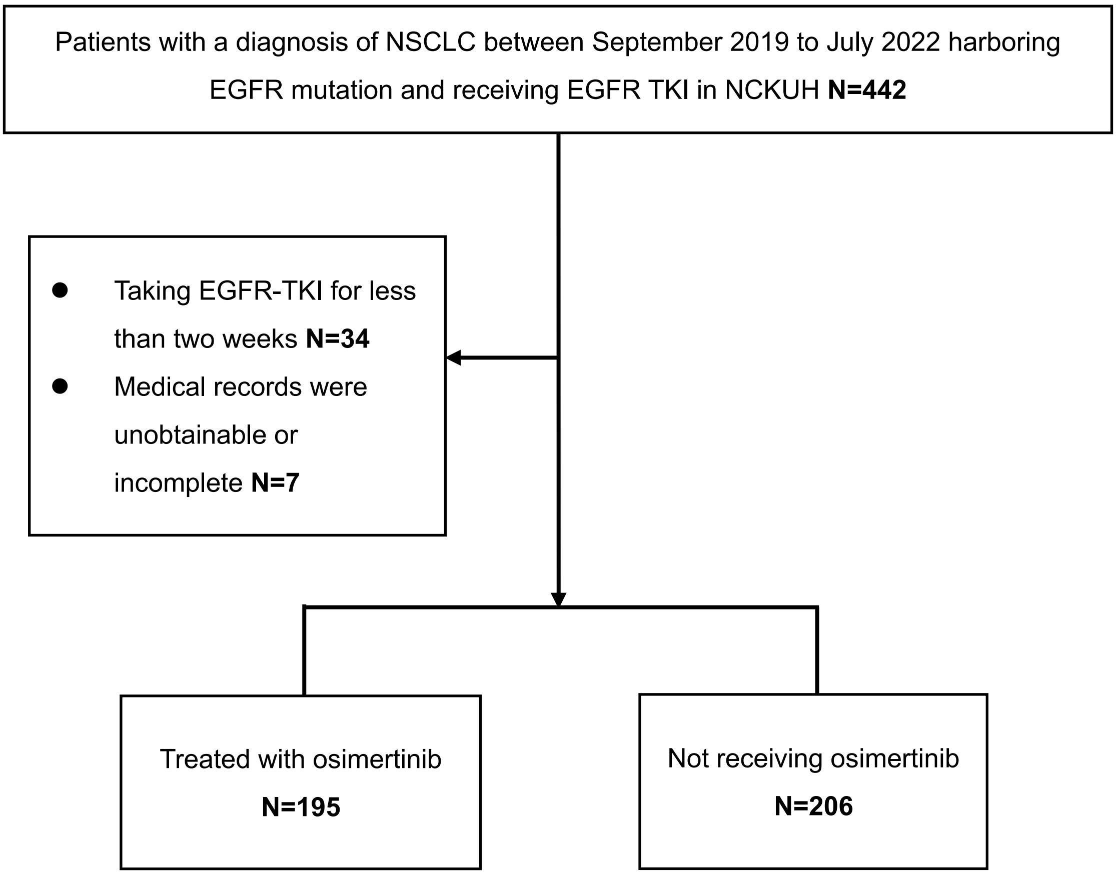

© 2024 Lin C-Y et al. *JAMA Network Open*.

eFigure 2. Accumulating Incidences of Cancer Therapy–Related Cardiac Events (CTRCEs) Among Patients With Non–Small Cell Lung Cancer

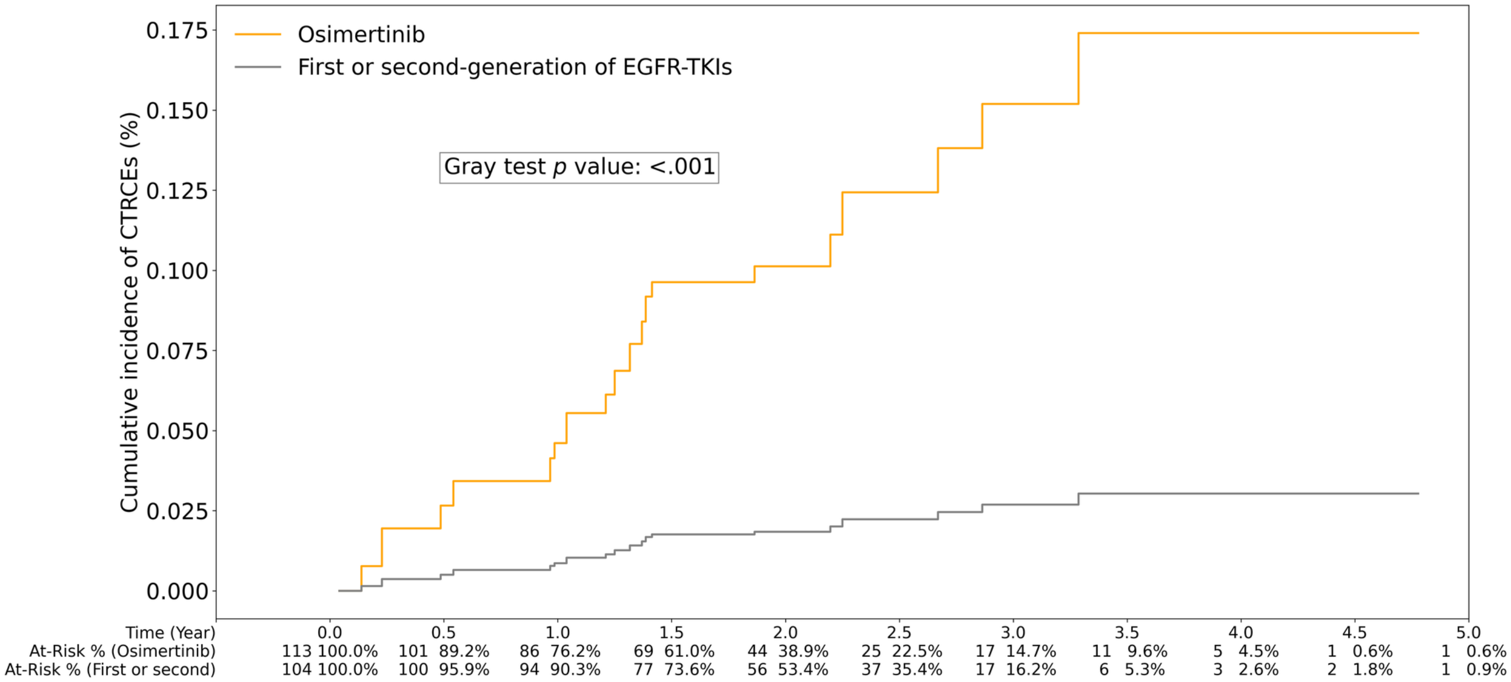

Patients had low pre-existing cardiovascular disease (CVD) or cardiovascular (CV) risk factors and were receiving osimertinib versus other epidermal growth factor receptor (EGFR) tyrosine kinase inhibitors (TKIs) (Gray test p-value = <.001)

© 2024 Lin C-Y et al. *JAMA Network Open*.

eFigure 3. Subgroup Analysis of Cancer Therapy–Related Cardiac Events (CTRCEs) Between Patients With Non–Small Cell Lung Cancers (NSCLC) Receiving Osimertinib vs Other Epidermal Growth Factor Receptor (EGFR) Tyrosine Kinase Inhibitors (TKIs)

Characteristic	Events Other vs Osi	Subdistribution HR (95% CI)	P value
Age >= 65	8 vs 19	0.35 (0.15-0.80)	.013
Age < 65	1 vs 10	0.11 (0.02-0.76)	.025
Male	3 vs 8	0.33 (0.09-1.18)	.089
Female	6 vs 21	0.24 (0.10-0.61)	.003
No TRT	8 vs 13	0.56 (0.23-1.34)	.193
No ACEI	6 vs 24	0.23 (0.09-0.55)	.001
No betablocker	6 vs 27	0.20 (0.08-0.49)	<.001
No antiplatelet	8 vs 29	0.24 (0.11-0.53)	<.001
No anticoagulant	8 vs 29	0.23 (0.10-0.51)	<.001
No statin	6 vs 24	0.21 (0.08-0.52)	<.001
No Arrhythmia drug	8 vs 28	0.24 (0.11-0.53)	<.001
No Arrhythmia	8 vs 26	0.27 (0.12-0.61)	.002
No DM	6 vs 22	0.24 (0.10-0.60)	.002
No HTN	2 vs 18	0.11 (0.03-0.43)	.002
No HL	6 vs 24	0.21 (0.08-0.52)	<.001
No CAD	7 vs 28	0.22 (0.10-0.51)	<.001
No CKD	8 vs 28	0.25 (0.12-0.56)	<.001
No HF	9 vs 29	0.27 (0.13-0.57)	<.001
No smoking	7 vs 25	0.24 (0.10-0.57)	.001
No alcohol	8 vs 28	0.25 (0.11-0.56)	<.001
BMI <24	6 vs 17	0.28 (0.11-0.71)	.007

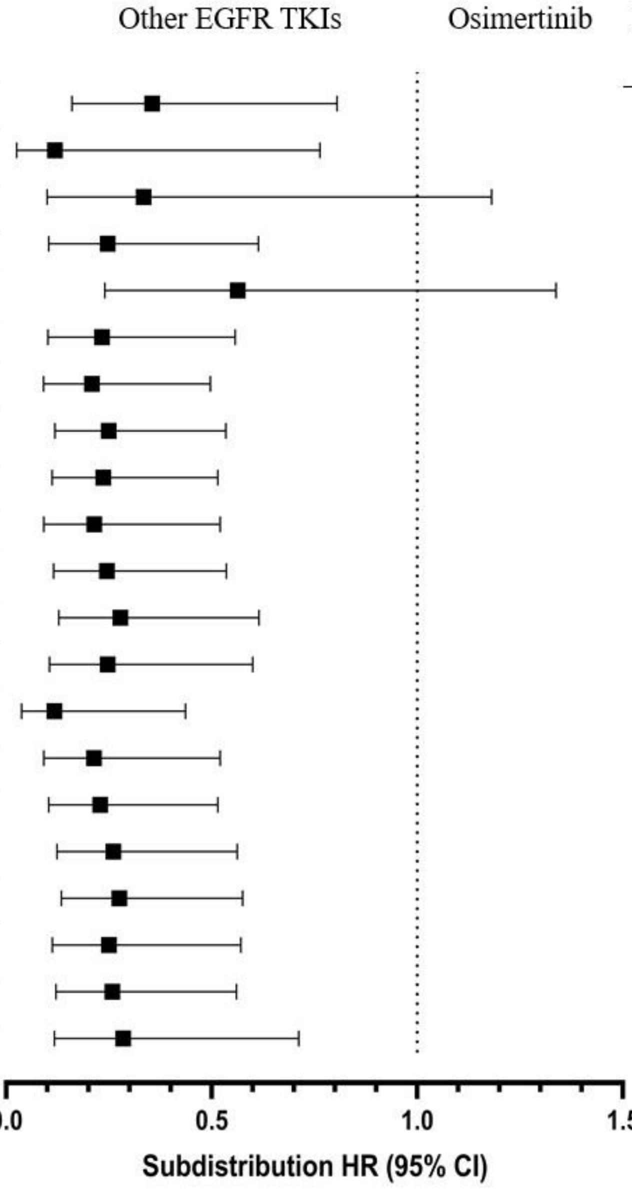

© 2024 Lin C-Y et al. *JAMA Network Open*.

eTable. Number and sHR of Individual CTRCEs of Patients With EGFR-Variant NSCLC Treated With or Without Osimertinib After IPTW

Patient, No (%)	Osimertinib use (N = 195)	Other EGFR-TKIs use (N =206)	sHR (95%CI)	*p*-Value
Newly developed arrhythmia	19	6	3.17 (1.32-7.59)	.01
Heart failure	8	2	3.87 (0.80-18.67)	.09
Valvular heart disease[a]	1	0	NA	NA
Myocardial infarction[a]	1	1	NA	NA

Abbreviations: CTRCEs, cancer therapy-related cardiac events; EGFR, epidermal growth factor receptor; HR, hazard ratio; IPTW, inverse probability of treatment weighting; NA, not applicable; NSCLC, non-small-cell lung carcinoma; sHR, subdistribution hazard ratio; TKI, tyrosine kinase inhibitor.

[a] The hazard ratio and p-value are not presented due to the small sample size.

© 2024 Lin C-Y et al. *JAMA Network Open*.
