# Supplementary material for: Cardiac Events and Survival in Patients With EGFR-Mutant Non–Small Cell Lung Cancer Treated With Osimertinib
Source: JAMA Netw Open. 2024 Dec 5;7(12):e2448364. doi: 10.1001/jamanetworkopen.2024.48364 (PMC11621985; doi:10.1001/jamanetworkopen.2024.48364)
Supplement: Supplement 2. — Data Sharing Statement [file jamanetwopen-e2448364-s002.pdf]

Data Sharing Statement

Lin. Cardiac Events and Survival in Patients With EGFR-Mutant Non–Small Cell Lung Cancer Treated With Osimertinib. *JAMA Netw Open*. Published December 05, 2024. doi:10.1001/jamanetworkopen.2024.48364

Data
Data available: No

Additional Information
Explanation for why data not available: De-identified patient data can be obtained through a reasonable request to the corresponding author, subject to approval by the Institutional Review Board of National Cheng Kung University Hospital and payment of processing fees.
